# 25-Hydroxycholecalciferol Serum Level Shows an Inverse Relationship with High-Grade Uterine Cervical Dysplasia in HIV-Uninfected Black Women in South Africa

**DOI:** 10.3390/jcm14113817

**Published:** 2025-05-29

**Authors:** Rivak Punchoo, Greta Dreyer, Tahir S. Pillay

**Affiliations:** 1 Department of Chemical Pathology, Faculty of Health Sciences, University of Pretoria, Pretoria 0001, South Africa; tahir.pillay@up.ac.za; 2 Division of Chemical Pathology, Department of Pathology, University of Cape Town, Cape Town 7701, South Africa; 3 Department of Obstetrics and Gynaecology, Faculty of Health Sciences, University of Pretoria, Pretoria 0001, South Africa; greta.dreyer@up.ac.za; 4 Tshwane Academic Division, National Health Laboratory Service (NHLS), Pretoria 0001, South Africa

**Keywords:** 25-hydroxycholecalciferol, cervical dysplasia, high-grade squamous intraepithelial lesion, HSIL, HIV, low-grade squamous intraepithelial lesions, LSIL, vitamin D

## Abstract

**Background:** Cervical dysplasia is a pre-malignant condition of the uterine cervix and is highly prevalent in Sub-Saharan Africa; especially affecting HIV-infected Black women. The anti-dysplastic effect of vitamin D hormones in cervical dysplasia is poorly understood. Therefore, we conducted a cross-sectional case–control observational study to assess the relationship between serum 25-hydroxycholecalciferol (25(OH)D) and cervical dysplasia, amongst Black women with and without HIV infection. **Methods:** The study participants attended a gynaecologic oncology clinic at an academic hospital in Pretoria, South Africa (*n* = 109). Patient clinical data were obtained during consultation. Cervical dysplasia was identified by cytology (PAP smear) which classified the case group as high-grade squamous epithelial lesions (HSILs), and the control group as <HSIL. Serum biochemistry measured 25(OH)D and its covariate biochemical variables. The data were statistically modelled to adjust for clinical and biochemical covariates, identify a significant relationship (*p* ≤ 0.05) between 25(OH)D and cervical dysplasia, and analyse subgroup interaction between HIV status and cervical dysplasia. **Results:** The data showed high levels of vitamin D insufficiency and deficiency in Black women with and without HIV infection. After covariate adjustment, 25(OH)D demonstrated an inverse relationship with HSIL in HIV-uninfected Black women. Furthermore, an interaction effect between women with and without HIV infection was observed. **Conclusions:** The role of 25(OH)D in the primary prevention of cervical dysplasia in Black women without HIV infection is promising, and dosing strategies require investigation. Also, future studies exploring the immunomodulatory role of 25(OH)D in cervical dysplasia in HIV-infected women is warranted.

## 1. Introduction

Cervical cancer exerts a global health burden, especially in resource-constrained countries [1]; for example, cervical cancer constituted 21.7% (*n* = 12 983) of new cancer cases in South Africa in 2018 [2]. The Human Papillomavirus (HPV), especially the oncogenic types of HPV, is the principal biological agent of cervical pre-cancerous lesions and cervical cancer [3]. Upon the entry of HPV into the uterine cervical epithelium, the virus persists in the cytoplasm or intercalates with human DNA, upregulating oncoprotein expression, which dysregulates tumour suppressor genes, causing pre-malignant dysplastic lesions of the cervical epithelium [3,4,5].

The Bethesda cytological classification identifies low-grade squamous epithelial lesions (LSILs), high-grade squamous epithelial lesions (HSILs), and other less common dysplastic lesions [6]. LSILs usually clear in most patients; however, HSILs demonstrate a high potential to transform into cervical cancer [7]. The key determinants of HSIL progression and cervical cancer are HPV oncogenic type and persistent HPV infection [8]. Abnormal cervical screening tests by cervical cytological assessment and molecular-based HPV assays are histologically assessed by cervical biopsy. Dysplastic changes observed in cervical biopsies are graded into three categories of cervical intraepithelial neoplasia (CIN): mild dysplasia (CIN 1), moderate dysplasia (CIN 2), and severe dysplasia or carcinoma-in situ (CIN 3). CIN 3 is considered a pre-malignant condition that can progress to cervical cancer and correlates with HSIL cytology.

The incidence and prevalence of HSILs, CIN of all grades, and cervical cancer increase in HIV infection [9,10,11,12,13,14]. HPV co-infection is more persistent in women with HIV infection [15], and immune biomarker levels influence risks and CIN grade [16]. Cervical cancer is defined as an AIDS-defining condition [17,18], and the early initiation of highly active antiretroviral treatment (HAART) with sustained adherence, limits progression to CIN and the incidence of invasive cervical cancer [19].

Pre-clinical studies that have investigated the anti-cancer action of early vitamin D precursors (cholecalciferol and 25-hydroxycholecalciferol, 25(OH)D) and the fully activated hormone (calcitriol, 1.25-dihydroxycholecalciferol) in cervical cancer have shown chemotherapeutic promise [20,21,22,23,24]. These pre-clinical studies have demonstrated a growing body of data showing that the vitamin D metabolome exerts anti-cancer actions by numerous intracellular mechanisms, including anti-inflammation, apoptosis, cell cycle arrest, and anti-angiogenesis [25,26,27].

There are limited clinical studies describing the effect of serum 25(OH)D, the currently recommended biomarker of vitamin D status [28], in early cervical tumorigenesis. The clinical studies that have investigated the effect of 25(OH)D levels in cervical pre-malignant lesions on the outcomes of dysplastic cervical grade and HPV clearance demonstrate conflicting results [29,30]. Furthermore, studies exploring the relationship between 25(OH)D in Black women in Africa and HIV infection in cervical tumorigenesis are neglected research topics. However, these populations are at risk of vitamin D deficiency from traditional and HIV-related factors [31,32]. Thus, clinical studies have currently identified inconclusive evidence to support the role of vitamin D in cancer prevention and treatment [33]. Therefore, formal clinical guidelines for primary and secondary cancer prevention and treatment are unspecified [34].

The assessment of 25(OH)D levels in cervical pre-malignancy conditions is also complicated by the variable cut-off points based on optimal bone health outcomes and debated amongst leading international clinical societies [35]. For example, the Endocrine Society Task Force [26] defines clinically relevant categories of vitamin D as sufficiency (25(OH)D > 30 ng/mL [>75 nmol/L]); insufficiency (25(OH)D: 21–29 ng/mL [52.5–72.5 nmol/L]), and deficiency (25(OH)D < 20 ng/mL [<50 nmol/L]) [28] and contrasts to the lower cut-off points by the Institute of Medicine (IOM) [33,36].

In order to understand the impact of vitamin D on cervical dysplastic progression in African women, 25(OH)D body reserve status was evaluated in women who demonstrated abnormal cervical cytology showing advanced cervical dysplasia (HSIL) and benign cervical dysplasia (<HSIL). Owing to the direct negative impact of HIV on the progression of cervical dysplasia, the anti-dysplastic relationship of 25(OH)D on African women both with and without HIV infection was evaluated. Therefore, this study employed a case–control analysis to model the relationship between serum 25(OH)D in HSIL cases and <HSIL controls in cervical dysplasia groups of Black women with and without HIV infection.

## 2. Results

### 2.1. Characteristics of the Study Population

The sample demographic data of participants are listed in Table 1.

### 2.2. Biomarker Summary Statistics of 25(OH)D and Covariate Serum Analyte Parameters Affecting 25(OH)D Levels

A summary of 25(OH)D and covariate serum analytes impacting 25(OH)D levels in the women with and without HIV infection with cervical dysplasia is listed in Table 2. Notably, the 25(OH)D levels (with and without seasonal adjustment) were below the sufficiency cut-off point of 30 ng/ml in HIV-infected and HIV-uninfected women.

### 2.3. Clinical Classification of Vitamin D Status

The clinical classification of vitamin D status is elaborated in Table 3, which indicates three categorical levels of vitamin D status classified by the Endocrine Society Task Force [28].

### 2.4. Evaluating the Relationship Between Serum 25(OH)D and Cervical Dysplasia Case and Control Groups in Women with and Without HIV Infection

#### 2.4.1. Evaluation of Clinical and Biochemical Parameters Associated with the Main Effects of Cervical Dysplasia and HIV Status

The clinical parameters age, height, mass, and body mass index (BMI) and the biochemical parameters serum creatinine, eGFR, serum corrected calcium, albumin, serum magnesium, serum phosphate, parathyroid hormone, and 25(OH)D were evaluated for their association with the main effects of cervical dysplasia (HSIL and <HSIL) and HIV status. Significant interactions were observed with the outcome variables age (*p* = 0.038) and 25(OH)D (*p* = 0.013) (Table S1, Supplementary Data).

The mean age for patients with HSIL differed from those with <HSIL by 0.61 years and was insignificant (*p* = 0.780). In contrast, a subgroup analysis in patients with HIV infection identified a marginally significant difference in mean age (5.42, 95% CI −0.79–11.63; *p* = 0.087). The interaction between HSIL and HIV status was significant (Table S1; *p* = 0.038) and indicates that the mean age of women with and without HIV infection changed in opposing directions in the cervical dysplastic categories.

The mean 25(OH)D levels in women with HIV between controls vs. cases showed marginal significance with cervical dysplasia (Table S1; *p* = 0.061). The interaction between cervical dysplasia and HIV status was significant (*p* = 0.013) and suggests that lower levels of 25(OH)D level predict progression to HSIL in women without HIV compared to women with HIV infection.

#### 2.4.2. Adjustment for 25(OH)D by Clinical and Biochemical Parameters

Clinical and biochemical parameters were assessed for the confounding effects on 25(OH)D (Table S2, Supplementary Data), and notably, parathyroid hormone (PTH) showed a significant effect on 25(OH)D (*p* = 0.004). This identifies an effect modifier property where the variable interacts with the risk factor and, in this instance, shows a physiological homeostatic relationship. This effect-modifying action of PTH changes the relationship between 25(OH)D on the main effects of cervical dysplasia and HIV status. There were no significant confounding effects by other variables (Table S2): BMI (*p* = 0.083), serum creatinine (*p* = 0.285), eGFR (*p* = 0.578), corrected calcium (*p* = 0.983), ALT (*p* = 0.848), serum magnesium (*p* = 0.205), and serum phosphate (*p = 0*.469).

#### 2.4.3. Final Model and Analysis of 25(OH)D as a Function of Cervical Cytology, HIV Status, Interaction Term, and Adjustment for PTH

**Figure 1:**
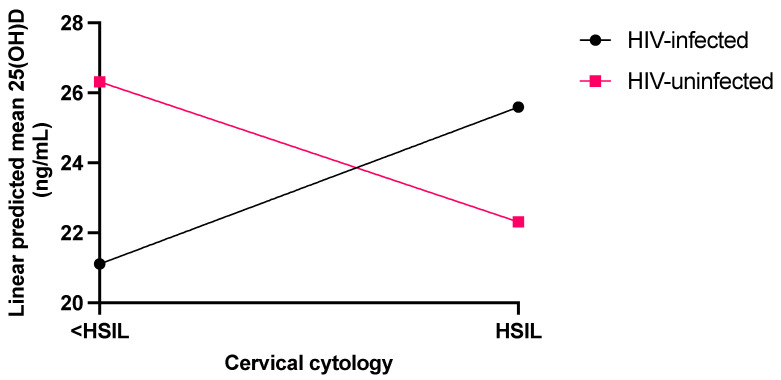
Linear predicted means of 25(OH)D adjusted for PTH covariate in women with and without HIV infection in the cervical dysplasia categories of <HSIL (control) and HSIL (cases).

The final model (Figure 1), adjusted for the modifying effect of PTH, showed that the interaction between cervical dysplasia and HIV status was significant (*p* = 0.005). In particular, for women without HIV, protective anti-dysplastic action to high-grade cervical dysplasia was observed with a mean 25(OH)D of 22.31 (95% CI: 19.25–25.37) ng/mL compared to 26.31 (95% CI: 23.64–29.00) ng/mL for <HSIL. In contrast, women with HIV infection had an increased cervical dysplastic effect with a mean 25(OH)D level of 25.59 (95% CI: 22.58–28.59) ng/mL compared to 21.11 (95% CI:18.18–24.05) ng/mL for <HSIL.

## 3. Discussion

The high incidence and prevalence of cervical cancer in Sub-Saharan Africa emphasises the need for alternative approaches such as nutritional support to promote the clearance of uterine cervical HPV infection [37]. Pre-clinical studies demonstrate the anti-cancer action of calcitriol and precursor vitamin D metabolites in cervical cancer [20,21,22,23,24,25] which complements other chemotherapeutic interventions [26]. However, the potential anti-dysplastic action of endogenous vitamin D metabolites in uterine cervical dysplasia is limited, especially amongst Black women and in HIV infection. Therefore, our case–control study modelled 25(OH)D serum levels (adjusted for clinical and biochemical confounders and covariates) in HSIL cases and a control group (<HSIL) in Black women with and without HIV infection in South Africa.

This study classified the majority of participants in both groups with and without HIV infection into clinically insufficient or deficient 25(OH)D categories based on the Endocrine Society Task Force’s 25(OH)D cut-off point recommendations [28]. Black women are reported to have lower vitamin D reserves than White women in the USA [38], owing to their darker pigmented skin, which impairs the activation of 7-dehydrocholesterol skin precursor to cholecalciferol [39]. In the group with HIV infection, traditional risk factors and HIV antiretroviral therapy may further contribute to an insufficient or deficient 25(OH)D status [40]. Despite the lower 25(OH)D status, Black females demonstrate lower bone turnover, bone loss, fractures, and higher calcium absorption and retention [41]. Bone regeneration and formation are dependent on the host immunity and metabolic factors, and close inter-dependence with electroactive and electrosensitive components of bone tissue, together with bone matrix components [42,43,44].

The optimal cut-off points for extra-skeletal anti-cancer and immunomodulatory actions for 25(OH)D are currently unavailable [28]. The effect-modifying action of PTH in this study reflects the negative physiological feedback by low 25(OH)D on the parathyroid hormone secretion [42] and is observed in Black females [43]. Also, skeletal resistance to PTH in Black women and improved calcium retention [44] may account for the corrected calcium level within the reference interval. PTH secretion and action may also be impaired in HIV infection and additionally be affected by HAART regimen [45], and, thus, the effect-modifying action of PTH in HIV infection additionally requires the consideration of non-traditional factors that affect PTH secretion and action.

In this study, the women not infected with HIV showed an inverse relationship between HSIL and 25(OH)D serum levels, suggesting that 25(OH)D decreased progression from <HSIL to HSIL. In addition, there was an interaction effect between women with HIV and women without HIV infection, where it was observed that the women with HIV showed an unexpected direct relationship between HSIL and serum 25(OH)D levels, suggesting that 25(OH)D was not protective against progression to HSIL in this group.

Many studies support our finding of an inverse relationship between 25(OH)D serum level and HSIL in women without HIV. In a cross-sectional study (*n* = 2353) by the National Health and Nutrition Examination Survey (NHANES), 2003–2006, the association between 25(OH)D (as continuous and clinical categorical variables) and cervicovaginal HPV infection showed that the adjusted odds ratio for serum 25(OH)D conferred protection against HPV infection. Each 10 ng/mL decrease in 25(OH)D level increased HPV infection risk (adjusted odds ratio, 1.14; 95% CI, 1.02–1.27). Furthermore, the authors demonstrated that the odds of acquiring HPV infection amongst HPV-vaccinated women were increased at levels of serum 25(OH)D lower than the sufficient cut-off point of ≥30 ng/mL [46]. A recent follow-up study by Gupta et al. [47] examined the NHANES data from 2009 to 2014 and showed that 25(OH)D deficiency (<20 ng/mL) was significantly associated with low-risk and high-risk HPV (hrHPV) cervical infection (RR 1.41, CI 1.23–1.61, *p* < 0.001; RR 1.25, CI 1.04–1.49, *p* = 0.014, respectively). This finding is consistent with a study in mid-adult women in the USA that showed a significant positive association between multiple serum vitamin D biomarkers and short-term persistence of fourteen high-risk HPV types [29]. Furthermore, a case–control study amongst Turkish women observed that the mean serum 25(OH)D was significantly lower (*p* = 0.009) in women with HPV infection with abnormal cytological smears [48]. Collectively, these studies are consistent with our observation of an inverse relationship between 25(OH)D serum level and HSIL in women without HIV infection and invite further investigation by longitudinal 25(OH)D supplementation studies in various patient sub-populations to clarify the protective action of 25(OH)D in cervical dysplasia.

In contrast, other studies show contrary findings to our observation, describing the modest or absent relationships between 25(OH)D serum level and HSIL in women without HIV infection. These studies show variable observational designs, patient demographics, and ethnic inclusion, which may impact 25(OH)D’s association with HPV clearance and protective effects against cervical dysplastic progression. The HITCH Cohort Study enrolled 18- to 24-year-old sexually active university students in Montreal, Canada, and showed only a modest negative association with HPV clearance with every 10 ng/mL increase in serum 25(OH)D level (hazard ratio [HR], 0.76; 95% CI, 0.60–0.96) and a marginal association between HPV clearance and 25(OH)D levels <30 ng/mL compared to >30 ng/mL (odds ratio [OR], 2.14; 95% CI, 0.99–4.64) [49]. Another study investigating multiple vitamin D biomarkers did not identify a significant association between 25(OH)D and other serum biomarkers of vitamin D metabolism with hrHPV prevalence in women between 30 and 50 years old in the USA. However, the catabolic 25(OH)D product, 24,25-dihydroxycholecalciferol (24,25(OH)_2_D3), showed a significant association with higher odds of hrHPV infection [30]. Additionally, in a case–control study in an endemically vitamin D-deficient region in Turkey, the association between 25(OH)D and hrHPV infection types (*n* = 49) with HPV-negative control (*n* = 94) did not identify a significant change in serum 25(OH)D as a continuous variable (*p* = 0.774) or as a clinical classifier of 25(OH)D status (*p* = 0.989) [50]. The variability in study results supporting the protective role of 25(OH)D in cervical dysplasia reveals variation in study design, sample size, and the inclusion of diverse ethnic participant groups. Furthermore, it would be interesting to consider the variance between studies by investigating the autocrine vitamin D metabolism in cervical dysplastic lesions to elucidate intracellular vitamin D metabolism and HPV clearance.

There has been limited investigation of 25(OH)D nutritional supplementation on the regression of cervical dysplastic lesions. In a randomised, double-blind, placebo-controlled trial of Iranian patients with CIN 1 (*n* = 29 placebo group and *n* = 29 experimental group), supplementation with 50 000 IU vitamin D3 twice weekly for six months showed significant CIN 1 regression in participants who received vitamin D3 supplementation (84.6% vs. 53.8%, *p* = 0.01) [51]. In a case–control study amongst Japanese women, an insignificant association between dietary vitamin D intake evaluated by a semiquantitative food frequency questionnaire and CIN3 risk was observed (*p* for trend = 0.109) [52]. This study was limited by modest sample size, recall bias, and the omission of serum 25(OH)D measurement. A noteworthy observation from these two studies is the potential variation in ethnic characteristics that may mediate the protective effect of 25(OH)D supplementation in cervical dysplasia. Furthermore, longitudinal studies need to establish the protective role and dosing requirements to maintain 25(OH)D at optimal therapeutic levels to enhance cervical HPV clearance and progression to HSIL.

The significant interaction effect between 25(OH)D levels in women with and without HIV infection and cervical dysplasia was unexpected and suggests an altered immune regulation by 25(OH)D in women with HIV infection. HPV is efficient at evading host detection by blunting the adaptive immune arm, resulting in HPV persistence with an increased probability of progressing to HSIL [53]. The T-helper CD4+ lymphocyte is critical to mounting effective cell-mediated immunity (CMI). Persons with HIV infection, even on HAART, show an incomplete restitution of the CD4+ T cell population [54] and, therefore, HPV persistence and early progression to cervical cancer [55,56]. Inflammation contributes to the development and progression of cancer mediated by pro-inflammatory proteins by activating tumorigenic signalling pathways [57,58,59,60]. In contrast, calcitriol demonstrates anti-inflammation properties via various intracellular mechanisms that regulate the intrinsic and adaptive arms of the immune system via vitamin D receptor upregulation in most immune cells [27,61]. Our findings postulate that higher 25(OH)D serum levels cause an incomplete and sub-optimal CMI response owing to an impaired CD4+ cell population in Black women with HIV infection, thus paradoxically triggering a chronic pro-inflammatory tumour microenvironment, viral persistence, and dysplastic progression to HSIL. Clinical studies in a large cohort with HIV infection examining inflammation pathways and inflammatory biomarkers in cervical dysplasia may assist in elucidating immune mechanisms which mediate our study’s observation.

The strengths of our study include investigating 25(OH)D’s anti-dysplastic action in a population of women who show a high prevalence of cervical dysplasia (i.e., Black women with HIV infection), which is poorly investigated in Africa. Therefore, we studied the anti-dysplastic action of 25(OH)D in Black women, who have a high prevalence of cervical dysplasia in Sub-Saharan Africa. We further included an important population subgroup consisting of women with HIV infection who demonstrate poor HPV clearance, persistent cervical dysplasia, and rapid progression to cervical cancer compared to women without HIV infection. In addition, the adjustment for relevant clinical and biochemical confounding variables yielded an accurate and valid model to describe the relationship between 25(OH)D and cervical dysplasia. This study, however, was limited by the lack of genotyping HPV types, which may have clarified the relationship between oncogenic HPV types, cervical dysplastic grade, and 25(OH)D. Furthermore, the cross-sectional sampling limited our understanding of 25(OH)D’s role in the dynamic clearance of cervical HPV infection and attributing causation. The latter limitation is inherent to case–control observational studies, and, therefore, this study provides an exploratory relationship of 25(OH)D’s anti-dysplastic action in cervical cancer.

## 4. Materials and Methods

### 4.1. Study Setting

The study was conducted at the Gynaecologic Oncology Clinic at Steve Biko Academic Hospital, a public tertiary academic hospital in Pretoria, Gauteng, South Africa. Black African female patients from peripheral health clinics were referred to this hospital with abnormal cervical cytology results (<HSIL and HSIL) for consultation regarding histological confirmation and treatment.

### 4.2. Study Population

#### 4.2.1. Size of Study Population

This study was conducted within a factorial study design with the main effects of HIV status (positive or negative) and cervical cytology status (HSIL or <HSIL). Data analysis employed a two-way analysis of variance, or an appropriate linear regression, with the two main effects and their interaction. The sample size for this scenario was generally regarded as adequate when the residual mean square degrees of freedom was at least thirty, i.e., at least nine patients for each HIV status–cytology combination. However, since estimates within HIV status cytology combination groups were also of interest, a sample size of approximately twenty-five patients per group was the aim. The interpretation of statistical significance also considered clinical relevance.

#### 4.2.2. Enrolment of Study Participants

Briefly, patients attended a clinical consultation, and patients who met the inclusion and exclusion criteria for participation in this study consented as outlined in the institutional ethics clearance informed consent form.

During the consultation, medical history was recorded, and a registered health professional reviewed the clinical information. All patients enrolled for participation met the inclusion and exclusion criteria of the study and signed an informed consent form. Women included in the study were Black and HIV-suppressed on highly active antiretroviral drug (HAART) regimens adherent to the South African state sector clinical guidelines [62].

Women with a history of cancer or the consumption of nutritional supplementation with calcium or vitamin D, tobacco smokers, and those taking anti-epileptic and oral contraceptives were excluded from the study. Patients with renal and liver disease were also excluded from participation.

All patients had their height (m) and mass (kg) measured, and their body mass index (BMI) was calculated using the equation: *BMI* = *mass* (*kg*)/ℎ*eig*ℎ*t* (*m*)^2^. The patient’s history and laboratory data were obtained from history taking and clinical notes during the consultation and the laboratory information system, respectively.

The study was conducted according to the guidelines of the Declaration of Helsinki and approved by the Research Ethics Committee of the Faculty of Health Sciences, University of Pretoria (protocol code 31/2021, and date of approval 16 April 2021). All participants selected for inclusion in the study signed an informed consent form at enrolment.

### 4.3. Biochemical Analysis

All biochemical analytes were quantified at a private diagnostic South African laboratory (Ampath Laboratories, Pretoria, South Africa), which holds national laboratory accreditation. Assay kits for each analyte were purchased from the instrument manufacturer, the methods were validated, and quality was assured by running daily internal quality controls and subscribing to external quality assurance schemes, with all assays performing within acceptable precision and accuracy parameters. The parathyroid hormone was quantified by an electrochemiluminescent immunoassay on a Roche Cobas e411 instrument (Basel, Switzerland). The serum total 25(OH)D was quantified by a paramagnetic particle chemiluminescent immunoassay by a UNiCel Dxl Immunoassay System (Beckman Coulter, Brea, California, USA). As seasonal variation affects 25(OH)D levels [63,64], the measured 25(OH)D levels were seasonally adjusted for the Southern Hemisphere [65,66]. The serum phosphate was determined by a photometric UV method that uses molybdate to form a heteropolyacid complex with inorganic phosphate, analysed by spectrophotometric measurement at 340/380 nm on an AU Chemistry Analyser (Beckman Coulter, CA, USA). The serum creatinine was measured by the kinetic Jaffe uncompensated method traceable to the IDMS reference method, analysed on an AU Chemistry Analyser (Beckman Coulter, CA, USA), with the estimated glomerular filtration rate (eGFR) calculated by the Chronic Kidney Disease Epidemiology Collaboration (CKD-EPI) equation [67]. The serum total calcium was measured by a photometric test based on calcium ions reacting with Arsenazo III to form a purple-coloured compound, quantified bi-chromatically at 660/700 nm on an AU Beckman Coulter chemianalyser (Brea, CA, USA). The serum albumin was measured by the bromocresol green complexing to albumin and was spectrophotometrically assessed (600/800 nm) and used to adjust the serum calcium measurement by the following equation: *Adjusted* *calcium* = *Total* *measured* *calcium* + [(39.9 − *Albumin*) × 0.012], where albumin is in g/L and calcium in mmol/L. The serum alanine transaminase was analysed based on the International Federation of Clinical Chemistry (IFCC) recommendations using a kinetic UV two-step assay, which quantifies the consumption of NADH at 340 nm and is proportional to ALT activity and was performed on an AU Chemistry analyser (Beckman Coulter, CA, USA).

### 4.4. Statistical Analysis

A maximum likelihood regression model was employed to determine the relationship between the individual clinical and biochemical parameters and the fixed effects of cervical dysplasia (<HSIL; HSIL) and HIV status (uninfected; infected) and their interaction. The fitted model reported marginal means, 95% confidence intervals, and *p*-values (Supplemental Table S1). For 25(OH)D, the above model was also assessed using clinical and biochemical covariates. The covariates were included individually in the model (Supplementary Table S2), and those covariates that came up significant at the liberal significance level of 0.1 were then included together in the model. Only two covariates, BMI (*p* = 0.083) and PTH (*p* = 0.004), qualified for inclusion, and ultimately, only PTH remained in the model. All the data were analysed by StataCorp. 2021. Stata Statistical Software: Release 17. College Station, TX, USA: StataCorp LLC.

## 5. Conclusions

Our case–control study reveals that vitamin D insufficiency and deficiency states in Black South African women with and without HIV infection with cervical dysplasia are common. A model adjusting for confounding variables showed an inverse relationship between 25(OH)D level and the progression of <HSIL to HSIL in HIV-uninfected cases. In addition, the interaction between HIV-infected and HIV-uninfected status identifies a paradoxical relationship between 25(OH)D and high-grade cervical lesions in HIV infection and suggests a disruption of immune modulation in HIV infection. Future longitudinal observational studies can benefit from exploring inflammatory biomarkers and immune activation pathways in HIV infection to mechanistically clarify the role of 25(OH)D in cervical dysplastic progression. Furthermore, studies can investigate 25(OH)D supplementation to identify optimal dosing strategies to curb HSIL in Black women without HIV infection.

## Figures and Tables

**Table 1:** Characteristics of the study population.

	HIV-Uninfected (%, n)		HIV-Infected (%, n)	
**Cervical dysplasia**	**HSIL**	**<HSIL**	**HSIL**	**<HSIL**
**Age (years)** Mean age	42.67	46.30	43.60	38.17
(95% CI)	(38.23–47.11)	(42.44–50.16)	(39.24–47.95)	(33.74–42.61)
**Comorbidities**				
Hypertension (n)	4	11	4	2
Diabetes (n)	0	2	0	0
Arthritis (n)	0	1	0	0
Anaemia (n)	1	0	1	0
Asthma (n)	0	2	1	0
Hypercholesterolemia (n)	0	1	1	0
Peptic ulcer disease (n)	1	1	0	0
**Cervical dysplasia**				
Total	25	33	28	23
LSIL		8		8
ASCUS		12		6
ASC-H		12		8
AGC		1		1
HSIL	25		28	
**Mass (kg)** (mean ± SD)	78.69 (±7.07)	77.42 (±17.71)	69.49 (±11.42)	67.65 (±10.73)
**Height (m)** (mean ± SD)	1.62 (±0.08)	1.61 (±0.05)	1.57 (±0.11)	1.58 (±0.10)
**Body mass index (kg/m^2^),** (mean ± SD)	29.90 (±6.02)	30.00 (±6.86)	28.18 (±5.79)	28.06 (±6.92)

**Table 2:** Summary of serum analytes in case (HSIL) and control (<HSIL) groups.

Analyte (Serum)	HIV-Infected		HIV-Uninfected	
(Reference Interval)	HSIL	<HSIL	HSIL	<HSIL
**Calcium**	2.35 (±0.00)	2.40 (±0.12)	2.33 (±0.11)	2.31 (±0.11)
(2.15–2.50 mmol/L)				
**Calcium Corrected**	2.35 (±0.10)	2.38 (±0.11)	2.32 (±0.09)	2.31 (±0.08)
(2.15–2.50 mmol/L)				
**Albumin**	40.38 (±4.95)	42.13 (±2.39)	40.07 (±3.77)	41.00 (3.71)
(35–52 g/L)				
**Magnesium**	0.83 (±0.02)	0.83 (±0.08)	0.81 (±0.06)	0.82 (±0.07)
(0.66–1.07 mmol/L)				
**Phosphate**	1.03 (±0.13)	1.08 (±0.16)	0.99 (±0.22)	1.05 (0.19)
(0.78–1.42 mmol/L)				
**25(OH)D**	22.05 (±5.52)	26.01 (±7.51)	24.79 (±9.29)	21.48 (±7.00)
(Sufficiency: >30ng/ml)				
**25(OH)D (adjusted)**	23.28 (±4.24)	26.06 (±7.70)	26.79 (±9.16)	22.17 (±6.59)
(Sufficiency: >30 ng/ml)				
**PTH**	35.52 (±19.09)	35.86 (±12.30)	40.85 (±17.0)	33.17 (±9.51)
(15–65 pg/mL)				
**ALT**	17.85 (±0.71)	18.63 (±12.38)	21.29 (±7.12)	24.26 (±12.5)
(<50 U/L)				
**Creatinine** (64–104 μmol/L)	64.85 (±2.12)	62.34 (±11.14)	65.25 (±17.90)	66.78 (±16.08)
**eGFR** (>90 mL/min/1.73 m^2^)	113.45 (±1.41)	114.09 (12.51)	110.32 (±16.85)	106.96 (±23.16)

**Table 3:** Clinical classification of vitamin D status based on seasonally adjusted 25(OH)D.

	HSIL (n)	<HSIL (n)	TOTAL (n, %)
**HIV-uninfected women**			
Sufficient	5	10	15 (26%)
Insufficient	12	16	28 (49%)
Deficient	7	7	14 (25%)
**Total**	24 *	33	57
**HIV-infected women**			
Sufficient	12	2	14 (28%)
Insufficient	9	10	19 (37%)
Deficient	7	11	18 (35%)
**Total**	28	23	51

* One participant’s 25(OH)D level was not tested due to an administrative requisition error.

## Data Availability

The original contributions presented in the study are included in the article/Supplementary Materials; further inquiries can be directed to the corresponding author/s.

## Supplementary Materials

The following supporting information can be downloaded at https://www.mdpi.com/article/10.3390/jcm14113817/s1: Table S1: Evaluation of clinical and biochemical parameters associated with cervical dysplasia and HIV status; Table S2: 25(OH)D adjusted for each potential confounder variable.
