# Supplementary material for: 25-Hydroxycholecalciferol Serum Level Shows an Inverse Relationship with High-Grade Uterine Cervical Dysplasia in HIV-Uninfected Black Women in South Africa
Source: J Clin Med. 2025 May 29;14(11):3817. doi: 10.3390/jcm14113817 (PMC12156911; doi:10.3390/jcm14113817)
Supplement: Supplementary file 1 [file jcm-14-03817-s001.zip › jcm-3539518-supplementary.pdf]

Supplementary Materials

25-hydroxycholecalciferol serum level shows an inverse relationship with high-grade uterine cervical dysplasia in Black women without HIV infection in South Africa

Rivak Punchoo*, Greta Dreyer, Tahir S. Pillay

*** Correspondence:** rivak.punchoo@uct.ac.za

Supplementary Tables

Supplementary Table S1. Evaluation of clinical and biochemical parameters associated with cervical dysplasia and HIV status

**Parameter**	**Mean**	**95% Confidence Interval**	**P value**
**1. AGE**			
**Cervical dysplasia**			
<HSIL	42.50	39.57 - 45.42	
HSIL	43.10	39.98 - 46.22	0.780
**HIV status**			
HIV-	44.60	41.68 - 47.52	
HIV+	40.71	37.59 – 43.83	0.074
**Cervical dysplasia HIV-**			
<HSIL	46.30	42.44 – 50.16	
HSIL	42.67	38.23 – 47.11	0.227
**Cervical dysplasia HIV+**			
<HSIL	38.17	33.735 - 42.61	

HSIL	43.60	39.244 – 47.95	0.087
**Interactions between cervical dysplasia and HIV status**			
<HSIL and HIV-	46.298	42.44 - 50.16	
<HSIL and HIV+	38.17	33.74 - 42.61	
HSIL and HIV-	42.67	38.24 - 47.11	
HSIL and HIV+	43.59	39.24 - 47.95	0.038
**2. WEIGHT**			
**Cervical dysplasia**			
<HSIL	72.98	69.17 - 76.80	
HSIL	74.31	70.24 - 78.34	0.642
**HIV status**			
HIV-	78.02	74.21 - 81.83	
HIV+	68.58	64.51 - 72.65	0.001
**Cervical dysplasia HIV-**			
<HSIL	77.65	72.61 - 82.69	
HSIL	78.44	72.65 - 84.23	0.841
**Cervical dysplasia HIV+**			
<HSIL	66.68	61.89 - 73.47	
HSIL	69.61	63.93 – 75.28	0.641
**Interactions between cervical dysplasia and HIV status**			
<HSIL and HIV-	77.66	72.61 – 82.69	
<HSIL and HIV+	67.66	61.89 – 73.47	
HSIL and HIV-	78.44	72.65 – 84.23	
HSIL and HIV+	69.61	63.93 – 75.28	0.840
**3. HEIGHT**			

**Cervical dysplasia**			
<HSIL	1.59	1.57 – 1.61	0.620
HSIL	1.59	1.57 – 1.62	0.005
**HIV status**			
HIV-	1.61	1.59 – 1.63	
HIV+	1.57	1.55 – 1.59	0.704
**Cervical dysplasia HIV-**			
<HSIL	1.61	1.58 – 1.63	
HSIL	1.61	1.59 – 1.65	0.749
**Cervical dysplasia HIV+**			
<HSIL	1.57	1.54 – 1.60	
HSIL	1.58	1.55 – 1.61	
**Interactions between cervical dysplasia and HIV status**			
<HSIL and HIV-	1.61	1.58 – 1.64	
<HSIL and HIV+	1.56	1.54 – 1.60	
HSIL and HIV-	1.61	1.58 – 1.65	
HSIL and HIV+	1.57	1.55 – 1.61	0.977
**4. BODY MASS INDEX (BMI)**			
**Cervical dysplasia**			
<HSIL	29.02	27.35 – 30.68	
HSIL	29.17	27.39 – 30.95	0.904
**HIV status**			
HIV-	29.95	28.28 – 31.62	
HIV+	28.11	26.33 – 29.89	0.104
**Cervical dysplasia HIV-**			
<HSIL	29.97	27.76 – 32.17	

HSIL	29.93	27.39 – 32.47	0.983
**Cervical dysplasia HIV+**			
<HSIL	27.94	25.40 – 30.47	
HSIL	28.30	25.81 – 30.79	0.840
**Interactions between cervical dysplasia and HIV status**			
<HSIL and HIV-	29.97	27.76 – 32.18	
<HSIL and HIV+	27.94	25.40 – 30.47	
HSIL and HIV-	29.93	27.39 – 32.47	
HSIL and HIV+	28.30	25.82 – 30.79	0.872
**5. SERUM CREATININE**			
**Cervical dysplasia**			
<HSIL	63.72	59.63 – 67.84	
HSIL	65.74	61.36 – 70.13	0.512
**HIV status**			
HIV-	63.58	59.48 – 67.09	
HIV+	65.91	61.53 – 70.30	0.447
**Cervical dysplasia HIV-**			
<HSIL	62.09	56.66 – 67.52	
HSIL	65.28	59.04 – 71.52	0.450
**Cervical dysplasia HIV+**			
<HSIL	65.60	59.36 – 71.84	
HSIL	66.27	60.15 – 72.39	0.881
**Interactions between cervical dysplasia and HIV status**			
<HSIL and HIV-	62.09	56.66 – 67.52	
<HSIL and HIV+	65.60	59.36 – 71.84	

HSIL and HIV-	65.28	59.04 – 71.51	
HSIL and HIV+	66.27	60.15 – 72.39	0.681
**6. ESTIMATED GLOMERULAR FILTRATION RATE (EGFR)**			
**Cervical dysplasia**			
<HSIL	111.53	106.84 – 116.21	
HSIL	111.43	106.44 – 116.43	0.979
**HIV status**			
HIV-	113.87	109.18 – 118.55	
HIV+	108.77	103.77 – 113.77	0.145
**Cervical dysplasia HIV-**			
<HSIL	114.24	108.05 – 120.44	
HSIL	113.44	106.32 – 120.55	0.868
**Cervical dysplasia HIV+**			
<HSIL	108.44	101.32 – 115.56	
HSIL	109.15	102.18 – 116.13	0.888
**Interactions between cervical dysplasia and HIV status**			
<HSIL and HIV-	114.24	108.05 – 120.44	
<HSIL and HIV+	108.44	101.32 – 115.56	
HSIL and HIV-	113.44	106.32 – 120.56	
HSIL and HIV+	109.15	102.18 – 116.13	0.829
**7. CALCIUM (CORRECTED)**			
**Cervical dysplasia**			
<HSIL	2.34	2.32 – 2.37	
HSIL	2.34	2.31 – 2.37	0.958
**HIV status**			

HIV-	2.36	2.34 – 2.39	
HIV+	2.32	2.29 – 2.34	0.013
**Cervical dysplasia HIV-**			
<HSIL	2.37	2.34 – 2.40	
HSIL	2.35	2.31 – 2.39	0.500
**Cervical dysplasia HIV+**			
<HSIL	2.30	2.27 – 2.34	
HSIL	2.33	2.29 – 2.36	0.516
**Interactions between cervical dysplasia and HIV status**			
<HSIL and HIV-	2.37	2.34 – 2.40	
<HSIL and HIV+	2.31	2.27 – 2.35	
HSIL and HIV-	2.35	2.31 – 2.39	
HSIL and HIV+	2.33	2.29 – 2.36	0.350
**8. ALBUMIN**			
**Cervical dysplasia**			
<HSIL	41.61	40.69 – 42.53	
HSIL	40.15	39.16 – 41.14	0.034
**HIV status**			
HIV-	41.28	40.35 – 42.20	
HIV+	40.54	39.55 – 41.52	0.28
**Cervical dysplasia HIV-**			
<HSIL	42.12	40.90 – 43.34	
HSIL	40.32	38.92 – 41.72	0.058
**Cervical dysplasia HIV+**			
<HSIL	41.04	39.64 – 42.44	
HSIL	39.96	38.58 – 41.34	0.282

**Interactions between cervical dysplasia and HIV status**			
<HSIL and HIV-	42.12	40.89 – 43.34	
<HSIL and HIV+	41.04	39.63 – 42.44	
HSIL and HIV-	40.32	38.91 – 41.72	
HSIL and HIV+	39.96	38.58 – 41.34	0.601
**9. MAGNESIUM**			
**Cervical dysplasia**			
<HSIL	0.82	0.81 - 0.84	
HSIL	0.82	0.80 – 0.84	0.780
**HIV status**			
HIV-	0.83	0.81 – 0.85	
HIV+	0.82	0.80 – 0.84	0.409
**Cervical dysplasia HIV-**			
<HSIL	0.83	0.80 – 0.85	
HSIL	0.83	0.80 – 0.86	0.768
**Cervical dysplasia HIV+**			
<HSIL	0.82	0.79 – 0.85	
HSIL	0.81	0.78 – 0.84	0.467
**Interactions between cervical dysplasia and HIV status**			
<HSIL and HIV-	0.83	0.80 – 0.85	
<HSIL and HIV+	0.82	0.79 – 0.85	
HSIL and HIV-	0.83	0.80 – 0.86	
HSIL and HIV+	0.81	0.78 – 0.84	0.465
**10. PHOSPHATE**			
**Cervical dysplasia**			
<HSIL	1.07	1.03 – 1.12	

HSIL	1.00	0.95 – 1.05	0.040
**HIV status**			
HIV-	1.06	1.00 – 1.10	
HIV+	1.02	0.97 – 1.07	0.325
**Cervical dysplasia HIV-**			
<HSIL	1.07	1.02 – 1.14	
HSIL	1.03	0.96 – 1.10	0.358
**Cervical dysplasia HIV+**			
<HSIL	1.07	0.99 – 1.14	
HSIL	0.97	0.90 – 1.04	0.043
**Interactions between cervical dysplasia and HIV status**			
<HSIL and HIV-	1.07	1.02 – 1.14	
<HSIL and HIV+	1.06	0.10 – 1.14	
HSIL and HIV-	1.03	0.96 – 1.10	
HSIL and HIV+	0.96	0.90 – 1.04	0.401
**11. PARATHYROID HORMONE (PTH)**			
**Cervical dysplasia**			
<HSIL	36.04	32.37 – 39.70	
HSIL	36.78	32.86 – 40.70	0.787
**HIV status**			
HIV-	35.67	31.98 – 39.34	
HIV+	37.16	33.29 – 41.05	0.584
**Cervical dysplasia HIV-**			
<HSIL	36.37	31.41 – 41.32	
HSIL	34.88	29.33 – 40.41	0.694

**Cervical dysplasia HIV+**			
<HSIL	35.68	30.25 – 41.10	
HSIL	38.88	33.33 – 44.41	0.419
**Interactions between cervical dysplasia and HIV status**			
<HSIL and HIV-	36.37	31.41 – 41.32	
<HSIL and HIV+	35.68	30.26 – 41.10	
HSIL and HIV-	34.88	29.33 – 40.41	
HSIL and HIV+	38.88	33.34 – 44.41	0.392
**12. ALANINE TRANSAMINASE (ALT)**			
**Cervical dysplasia**			
<HSIL	21.30	18.71 – 23.90	
HSIL	19.15	16.38 – 21.93	0.268
**HIV status**			
HIV-	18.11	15.52 -20.71	
HIV+	22.77	20.00 – 25.54	0.016
**Cervical dysplasia HIV-**			
<HSIL	18.58	15.14 – 22.01	
HSIL	17.60	13.66 – 21.54	0.714
**Cervical dysplasia HIV+**			
<HSIL	24.40	20.46 – 28.34	
HSIL	20.92	17.06 – 25.79	0.217
**Interactions between cervical dysplasia and HIV status**			
<HSIL and HIV-	18.58	15.14 – 22.01	
<HSIL and HIV+	24.40	20.46 – 28.34	
HSIL and HIV-	17.60	13.66 – 21.54	

HSIL and HIV+	20.92	17.05 – 24.79	0.519
**13. 25-HYDROXYCHOLECALCIFEROL (25OHD)**			
**Cervical dysplasia**			
<HSIL	23.60	21.60 – 25.59	
HSIL	23.70	21.55 – 25.85	0.944
**HIV status**			
HIV-	24.13	22.11 – 26.15	
HIV+	23.10	20.97 – 25.24	0.492
**Cervical dysplasia HIV-**			
<HSIL	25.72	23.08 – 28.35	
HSIL	22.29	19.20 – 25.38	0.099
**Cervical dysplasia HIV+**			
<HSIL	21.22	18.20 – 24.26	
HSIL	25.28	22.30 – 28.25	0.061
**Interactions between cervical dysplasia and HIV status**			
<HSIL and HIV-	25.72	23.08 – 28.36	
<HSIL and HIV+	21.22	18.20 – 24.26	
HSIL and HIV-	22.29	19.20 – 25.38	
HSIL and HIV+	25.28	22.31 – 28.25	0.013

Supplementary Table S2. 25(OH)D adjusted for each potential confounder variable

Confounding variable	Predicted Mean	95% Confidence Interval	P value
**1. BODY MASS INDEX (BMI)**			**0.083**
**Cervical dysplasia**			
<HSIL	23.60	21.63 – 25.56	
HSIL	23.70	21.58 – 25.82	0.944
**HIV status**			
HIV-	24.29	29.29 – 26.28	
HIV+	22.92	20.81 – 25.04	0.360
**Cervical dysplasia HIV-**			
<HSIL	25.74	23.14 – 28.35	
HSIL	22.25	19.20 – 25.30	0.088
**Cervical dysplasia HIV+**			
<HSIL	21.19	18.20 – 24.18	
HSIL	25.31	22.38 – 28.24	0.054
**Interactions between cervical dysplasia and HIV status**			
<HSIL and HIV-	25.90	23.29 – 28.51	
<HSIL and HIV+	21.01	18.01 – 24.01	
HSIL and HIV-	22.41	19.36 – 25.47	
HSIL and HIV+	25.14	22.20 – 28.08	0.010
**2. CREATININE**			**0.285**
**Cervical dysplasia**			
<HSIL	23.64	21.65 – 25.63	
HSIL	23.65	21.50 – 25.79	0.993
**HIV status**			

HIV-	24.12	22.18 – 26.19	
HIV+	23.04	20.91 – 25.16	0.441
**Cervical dysplasia HIV-**			
<HSIL	25.77	23.15 – 28.40	
HSIL	22.20	19.12 – 25.29	0.084
**Cervical dysplasia HIV+**			
<HSIL	21.24	18.22 – 24.26	
HSIL	25.26	22.30 – 28.22	0.062
**Interactions between cervical dysplasia and HIV status**			
<HSIL and HIV-	25.84	23.21 – 28.47	
<HSIL and HIV+	21.18	18.16 – 24.19	
HSIL and HIV-	22.27	19.19 – 25.35	
HSIL and HIV+	25.20	22.23 – 28.16	0.011
**3. ESTIMATED GFR (eGFR)**			**0.578**
**Cervical dysplasia**			
<HSIL	23.59	21.60 – 25.59	
HSIL	23.70	21.56 – 25.86	0.940
**HIV status**			
HIV-	24.19	22.17 – 26.21	
HIV+	23.04	20.90 – 25.12	0.446
**Cervical dysplasia HIV-**			
<HSIL	25.72	23.08 – 28.35	
HSIL	22.29	19.20 – 25.38	0.098
**Cervical dysplasia HIV+**			
<HSIL	21.22	18.19 – 24.25	
HSIL	25.29	22.32 – 28.26	0.060

**Interactions between cervical dysplasia and HIV status**			
<HSIL and HIV-	25.78	23.13 – 28.42	
<HSIL and HIV+	21.15	18.12 – 24.19	
HSIL and HIV-	22.35	19.25 – 25.44	
HSIL and HIV+	25.22	22.25 – 28.20	0.012
**4. CALCIUM (CORRECTED)**			**0.983**
**Cervical dysplasia**			
<HSIL	23.60	21.60 – 25.60	
HSIL	23.70	21.55 – 25.86	0.945
**HIV status**			
HIV-	24.13	22.10 – 26.17	
HIV+	23.10	20.94 – 25.26	0.499
**Cervical dysplasia HIV-**			
<HSIL	25.72	23.07 – 28.37	
HSIL	22.29	19.18 – 25.39	0.100
**Cervical dysplasia HIV+**			
<HSIL	21.23	18.19 – 24.26	
HSIL	25.28	22.31 – 28.26	0.062
**Interactions between cervical dysplasia and HIV status**			
<HSIL and HIV-	25.72	23.03 – 28.41	
<HSIL and HIV+	21.22	18.15 – 24.29	
HSIL and HIV-	22.29	19.20 – 25.39	
HSIL and HIV+	25.28	22.30 – 28.26	0.013
**5. ALANINE TRANSAMINASE (ALT)**			**0.848**
**Cervical dysplasia**			

<HSIL	23.58	21.58 – 25.59	
HSIL	23.72	21.56 – 25.88	0.929
**HIV status**			
HIV-	24.16	22.12 – 26.20	
HIV+	23.06	20.90 – 25.23	0.467
**Cervical dysplasia HIV-**			
<HSIL	25.71	23.08 – 28.35	
HSIL	22.30	19.20 – 25.39	0.100
**Cervical dysplasia HIV+**			
<HSIL	21.20	18.16 – 24.24	
HSIL	25.30	22.32 – 28.29	0.060
**Interactions between cervical dysplasia and HIV status**			
<HSIL and HIV-	25.74	23.09 – 28.39	
<HSIL and HIV+	21.17	18.08 – 24.26	
HSIL and HIV-	22.32	19.21 – 25.44	
HSIL and HIV+	25.27	22.30 – 28.24	0.012
**6. MAGNESIUM**			**0.205**
**Cervical dysplasia**			
<HSIL	23.63	21.64 - 25.61	
HSIL	23.68	21.55 – 25.82	0.969
**HIV status**			
HIV-	24.21	22.21 – 26.21	
HIV+	23.03	20.91 – 25.15	0.429
**Cervical dysplasia HIV-**			
<HSIL	25.68	23.07 – 28.30	
HSIL	22.33	19.27 – 25.41	0.104

**Cervical dysplasia HIV+**			
<HSIL	21.32	18.31 – 24.34	
HSIL	25.19	22.23 – 28.14	0.073
**Interactions between cervical dysplasia and HIV status**			
<HSIL and HIV-	25.76	22.14 – 28.34	
<HSIL and HIV+	21.24	18.23 – 24.25	
HSIL and HIV-	22.41	19.33 – 25.49	
HSIL and HIV+	25.11	22.14 – 28.06	0.016
**7. PHOSPHATE**			**0.469**
**Cervical dysplasia**			
<HSIL	23.50	21.49 – 25.51	
HSIL	23.81	21.64 – 25.98	0.837
**HIV status**			
HIV-	24.08	22.06 - 26.09	
HIV+	23.16	21.02 – 25.29	0.538
**Cervical dysplasia HIV-**			
<HSIL	25.67	22.03 – 28.03	
HSIL	22. 36	19.27 – 25.45	0.111
**Cervical dysplasia HIV+**			
<HSIL	21.07	18.02 – 24.13	
HSIL	25.42	22.44 – 28.42	0.048
**Interactions between cervical dysplasia and HIV status**			
<HSIL and HIV-	25.61	22.96 – 28.26	
<HSIL and HIV+	21.14	18.11 – 24.17	
HSIL and HIV-	22.30	19.22 – 25.39	
HSIL and HIV+	25.50	22.47 – 28.52	0.011

**8. PARATHYROID HORMONE (PTH)**			**0.004**
**Cervical dysplasia**			
<HSIL	23.82	21.84 – 25.80	
HSIL	23.88	21.74 – 26.03	0.964
**HIV status**			
HIV-	24.47	22.45 – 26.49	
HIV+	23.18	21.07 – 25.27	0.384
**Cervical dysplasia HIV-**			
<HSIL	26.43	23.75 – 29.11	
HSIL	22.42	19.37 – 25.48	0.053
**Cervical dysplasia HIV+**			
<HSIL	20.99	18.05 – 23.92	
HSIL	25.46	22.47 – 28.46	0.037
**Interactions between cervical dysplasia and HIV status**			
<HSIL and HIV-	26.31	23.64 – 29.00	
<HSIL and HIV+	21.11	18.18 – 24.05	
HSIL and HIV-	22.31	19.25 – 25.37	
HSIL and HIV+	25.59	22.58 – 28.59	0.005
